# Identification of Jingmen tick virus (JMTV) in *Amblyomma testudinarium* from Fujian Province, southeastern China

**DOI:** 10.1186/s13071-022-05478-2

**Published:** 2022-09-27

**Authors:** Yuli Zhang, Zhenfeng Li, Zheng Pang, Zhen Wu, Zhijuan Lin, Guoyu Niu

**Affiliations:** 1 WeiFang Medical University, Weifang, 261053 China; 2 Department of Public Health, Gaomi People’s Hospital, Weifang, 261500 China; 3 Tianjin International Joint Academy of Biomedicine, Tianjin, 300457 China

**Keywords:** Jingmen tick virus, Ticks, *Amblyomma testudinarium*, Fujian Province

## Abstract

**Background:**

Jingmen tick virus (JMTV) is a newly discovered tick-borne virus that can cause disease in humans. This virus has been authenticated as being extremely widespread worldwide and as posing a significant threat to public health and safety.

**Methods:**

We collected 35 ticks belonging to two tick species from wild boars in Nanping, Fujian Province, China. JMTV-specific genes were amplified by qRT-PCR and nested PCR to confirm the presence of this pathogen.

**Results:**

More than one third of of all ticks collected (11/35) were positive for JMTV. Viral sequences were obtained from three of the JMTV-positive ticks, including the complete genomic sequence from one tick. This was the first time that JMTV was identified in the hard-bodied tick *Amblyomma testudinarium*. Phylogenetic analysis revealed that JMTV from Fujian Province shared > 90% identity with other isolates derived from China, but was distinct from those reported in France and Cambodia.

**Conclusions:**

JMTV is characterized by relatively low mutations and has its own local adaptive characteristics in different regions. Our findings provide molecular evidence of the presence of JMTV in an overlooked tick species from an area not unrecognized as being endemic. They also suggest that JMTV occupies a wider geographical distribution than currently believed and is a potential disease vector.

**Graphical abstract:**

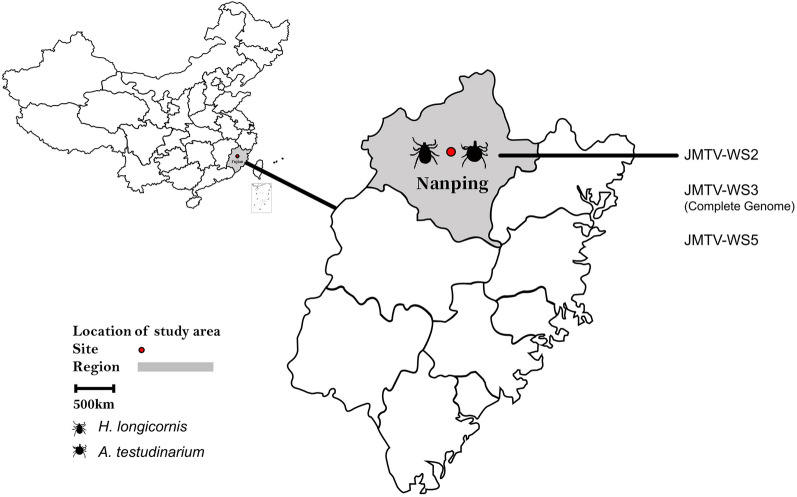

**Supplementary Information:**

The online version contains supplementary material available at 10.1186/s13071-022-05478-2.

## Background

Ticks are exclusively specialized obligate ectoparasites and are recognized as second only to mosquitoes in terms of transmitting various pathogens to human beings [1]. The main pathogens associated with ticks are protozoa, viruses and bacteria, all of which can survive and reproduce in different life stages of ticks and be transmitted through transovarial routes. In the past two decades, various tick-borne viruses (TBVs) have emerged across the world, attracting increasing attention from scientists and public health organizations. These emerging TBVs include severe fever with thrombocytopenia syndrome virus (SFTSV; 2011) and Alongshan virus (ALSV; 2019) in China [2, 3]; Heartland virus (HRTV; 2012) and Bourbon virus (BRBV; 2017) in the USA [4, 5]; Shibuyunji virus (2015) in Zambia [6]; Hunter island group virus (HIGV; 2015) in Australia [7]; and Muko virus (MUV; 2015), Tarumizu tick virus (TarTV; 2017) and Yezo virus (YEZV; 2021) in Japan [8–10]. At present, human-related TBVs involve five families: Nairoviridae, Phenuiviridae, Flaviviridae, Orthomyxoviridae and Reoviridae [11]. Viruses belonging to these families are transmitted from their natural hosts to humans through tick bites, causing symptoms of varying degree. Consequently, they have an adverse impact on public health.

Jingmen tick virus (JMTV) is one of the novel emerging TBVs that is as yet unclassified to any viral family or genus. It was first identified in the Asian blue tick *Rhipicephalus microplus* from the Jingmen region of Hubei Province, China, in 2014 [12]. The viral genome consists of four separate linear RNA segments: two of these encode nonstructural proteins (NSP1 and NSP2) that share homology with the NS5 and NS3 proteins of Flaviviridae viruses, respectively, and the other two segments code structural proteins that are as yet unmatched with any sequences currently available. These results suggest that JMTV may originate from the gene integration of an as-yet-undiscovered ancestral virus and Flaviviridae virus. Since its initial discovery in China, JMTV and JMTV-like viruses, which were defined as the Jingmenvirus group, have been identified in arthropods, cattle and monkeys sampled from Laos, Russia, Turkey, Kosovo, French Antilles, Uganda, Brazil and Trinidad and Tobago, as well as in bats and rodents from China [13–17]. More significantly, JMTV has been proven to infect humans and cause clinical symptoms, ranging from mild to severe [18]. Thus, it is of great urgency to strengthen entomological, virological and epidemiological research on JMTV in order to gain a better understanding of this virus and to be able to handle emerging related diseases.

Fujian Province is located in southeastern China, with mountains and hills accounting for > 80% of the total area of the province. It has the highest forest coverage rate of all provinces in China, with a forest area of 76.7 billion km^2^; as such, it is one of the six major forest areas in China [19]. Its abundant rainfall, sufficient light and superior climatic conditions make it suitable for plant growth and tick survival.

 In the study reported here, engorged ticks were collected from wild boars in the wild forest of Nanping region, Fujian Province. Molecular studies revealed the presence of JMTV nucleic acids in the tick samples, demonstrating the presence of this virus in southeastern China and, for the first time, its presence in the hard-bodied tick *Amblyomma testudinarium*. Comparison of the sequences of JMTV strains with those from other provinces in China revealed that the local strains showed evolutionary conservatism. The presence of this virus may pose threats and challenges to local public health security.

## Methods

### Tick collection and total nucleic acids preparation

**Fig. 1:**
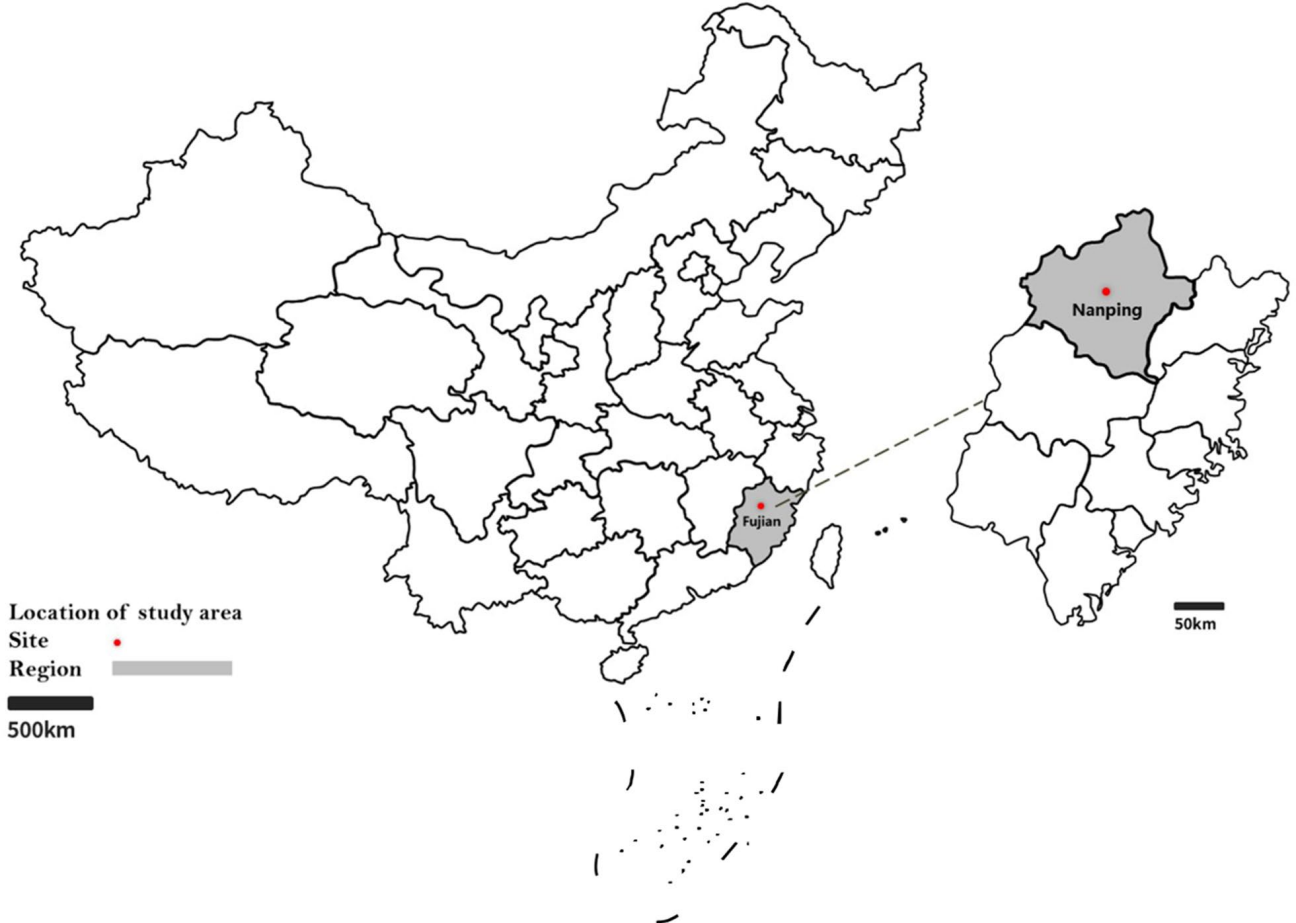
Location of Fujian Province in China (left) and the location of sampling site in the province where tick samples were collected, in 2019

During August 2019, ticks were collected from wild boars in the legal hunting ground of Nanping, Fujian Province (118°34′E, 27°83′N) (Fig. 1). The attached ticks were removed from the wild boars by forceps and stored individually in porous collection tubes containing moistened filter paper. The collected ticks were identified by morphology and then verified by the detection of the conserved tick gene cytochrome* c* oxidase I (*COI*). PCR primers used for *COI* PCR in this study were LCO1490 (GGT CAA CAA ATC ATA AAG ATA TTG G) and HCO2198 (TAA ACT TCA GGG TGA CCA AAA AAT CA). PCR amplification was performed in a 50-μl reaction volume containing 5 μl of extracted nucleic acids (extracted using Premix Taq [Takara Biomedical Technology Co., Ltd. China, Beijing, China]). The cycling conditions were: 94 °C for 5 min (initial denaturation); followed by 35 cycles of 94 °C for 30 s (denaturation), 55 °C for 1 min (annealing), 72 °C for 1 min (extension); with a final extension of 72 °C for 10 min. A negative control (no DNA template) was included. Successful amplification was determined subsequent electrophoresis in 1% agarose gel electrophoresis, and the electrophoresis products were visualized by SYBR® Safe stain (Thermo Fisher Scientific, Waltham, MA, USA). All ticks were maintained in a cool and ventilated place until analysis.

Tick specimens were individually homogenized in liquid nitrogen and centrifuged at 4 °C and 10,000 *g* for 10 min (Eppendorf, Hamburg, Germany) following the addition of 500 μl of cold Dulbecco's Modified Eagle Medium. The clarified supernatant of each tick homogenate was then prepared for RNA extraction using the QIAamp Viral RNA Mini Kit (Qiagen, Hilden, Germany), followed by complementary DNA (cDNA) synthesis using the Transcriptor High Fidelity cDNA Synthesis Kit (Roche Diagnostics Corp., Indianapolis, IN, USA) with random hexamer primer, as described by the manufacturer. The extracted and synthesized nucleic acids were stored at – 80 ˚C until PCR analysis.

### PCR for detection of JMTV in ticks

Quantitative reverse transcription PCR (qRT-PCR) was performed to confirm the presence of JMTV by amplifying the NS5-like protein gene located in genome segment 1. The qRT-PCR primer sets were JMTV-F (GTT GGC GGA GGA TGA AGA GAT) and JMTV-R (AAT CTC CCT CTG GAC CAC CAT), and the probe was JMTV-P (Texas Red-CCA CCA CCT CTA CCG GCT TTA CGC AG-BHQ2). qRT-PCR was performed using 5 µl of each aliquot of extracted RNA and the QuantiFast Probe RT-PCR Kit (Qiagen) following the manufacturer's instructions. The PCR cycling conditions were: 50 °C for 10 min, 95 °C for 5 min; then 40 cycles of 10 s at 95 °C and 30 s at 60 °C. The cycle threshold (Ct) value for a positive sample was set at 35 cycles, and a recombinant plasmid containing the target gene was used as the positive control. All positive specimens from the primary screening were subsequently tested by nested PCR using the primer sets Out-F (ATA GGC TGT CCA ACA CCG TGA T), Out-R (TGG ATC TCA TTG CCG TAC TTC AC) and In-F (TGG ATC TCA TTG CCG TAC TTC AC), In-R (GTA GAC CCT AGC CTC ATC TCC TCT) targeting the NSP1 gene. Nested PCR was performed with the same kit and conditions as for the *COI* gene described above. The amplified products were separated by electrophoresis in a 1% agarose gel electrophoresis, and the electrophoresis products were then visualized by SYBR® Safe (Thermo Fisher Scientific) and subjected to direct Sanger sequencing (Shanghai Sangon Biotechnology Co., Shanghai, China). The partial sequences of segment 1 obtained from the nested PCR have been deposited in GenBank database and assigned accession numbers (OM363694-OM363695).

### Genome sequencing

The AllPrep DNA/RNA mini Kit (Qiagen) was used to extract total DNA and RNA from the sample, according to the manufacturer’s instructions. Total RNA was subjected to library construction following a standard protocol provided by Illumina Inc. (San Diego, CA, USA). In brief, after removing ribosomal RNA from the sample, the remaining RNA was fragmented, reverse-transcribed, ligated to adaptors, purified and examined using the Agilent 2100 Bioanalyzer system (Agilent Technologies Inc., Santa Clara, CA, USA). Pair-end sequencing (150 bp) of the library was then performed on the HiSeq 2500 platform (Illumina Technologies Inc.). Raw reads were processed with quality checks and trimming, with open reading frame (ORF) prediction, and assembled de novo by MegaHit into contigs (> 200 bp). Acquired contigs were translated and aligned to the whole protein NCBI/NR reference database (October, 2021) by BLASTx. The contigs were mapped to the closely related sequences and checked for heterogeneity by visual inspection using UGENE v1.32.0 software(OM459837-OM459840).

The complete sequences of the JMTV genome were further confirmed by conventional PCR and Sanger sequencing with specific primers bracketing the missing sequences. The 5’ and 3’ end of each fragment was determined by rapid amplification of cDNA ends (RACE)-PCR analyses using a 5’/3’ RACE kit (Roche Diagnostics Corp.).

### Sequence analyses

Sequences obtained in this study were edited and aligned with SeqMan and MegAlign of the Lasergene software package(DNASTAR, Madison, WI, USA). Phylogenetic trees were inferred by using the neighbor-joining method available within MEGA7.0 software, with 1000 replicates with bootstrap values > 70% considered to be significant. Phylogenetic analyses of the nucleotides of the four segments of JMTV were restricted to the Jingmenvirus group. Complete nucleotide sequences of JMTV-related viruses were retrieved from GenBank. Additional phylogenetic analyses of JMTV-S1 partial sequences were compared with the amino acid sequences targeting the NS5-like gene of other Flaviviridae retrieved from GenBank. The accession numbers used in this study are shown in the relevant figures. These two phylogenetic analysis approaches generated a consistent topology. The NCBI conserved domain search tool (https://www.ncbi.nlm.nih.gov/Structure/cdd/wrpsb.cgi) was utilized to search the protein domain and important protein binding sites. Motif search was determined using MEME Suite 5.4.1 (https://meme-suite.org/meme/tools/meme).

### Virus isolation

Tick homogenates which were positive for JMTV viral RNA by qRT-PCR were centrifuged, and the supernatant was passed through a sterile 0.45-μm filter (MilliporeSigma, Burlington, MA, USA). The filtrates were then subjected to virus isolation using Vero and BHK–21 cells cultured in 6-well plates. Briefly, 100 μl of sample was inoculated onto a monolayer of cells at 37  C in 5% CO^2^ conditions. Cells were inspected daily and tested for the presence of JMTV RNA by qRT–PCR on days 6–8 post-infection. After three additional blind passages, the supernatants were harvested and cryopreserved at - 80˚C until further analysis. Virus isolation was performed at a BSL–2 laboratory in the School of Public Health, WeiFang Medical University.

## Results

### Tick collection and identification of JMTV

In total, 35 ticks of two tick species were collected from wild boar hosts, including *A. testudinarium* (*n* = 26 [74.3%], of which 23 were adults, 3 nymphs and 0 larvae) and *Haemaphysalis longicornis* (*n* = 9 [25.7%], of which 7 were adults, 2 nymphs and 0 larvae) (see Table 1). After identification, all attached ticks collected from the hosts were placed in a cool and ventilated place for 1 week and then transferred into liquid nitrogen. All ticks were treated individually in preparation for the detection of viral RNA.

**Table 1:** Detection of Jingmen tick virus viral RNA in ticks collected from wild boars, Fujian, China

Tick species		Tick ID	Animal/sex/age	Life of stage	qRT-PCR	Nested-PCR
*A. testudinarium*		WS-1	Wild boar 1/male/2–3 years	Adults	**−**	**/**
		WS-2	Wild boar 1/male/2–3 years	Adults	** + **	** + **
		WS-3	Wild boar 2/male/2–3 years	Adults	** + **	** + **
		WS-4	Wild boar 2/male/2–3 years	Nymphs	**−**	**/**
		WS-5	Wild boar 2/male/2–3 years	Adults	** + **	** + **
		WS-6 to WS-10	Wild boar 2/male/2–3 years	Adults	**−**	**/**
		WS-11	Wild boar 2/male/2–3 years	Nymphs	** + **	**−**
		WS-12	Wild boar 2/male/2–3 years	Adults	** + **	**−**
		WS-13 to WS-17	Wild boar 3/male/2–3 years	Adults	**−**	**/**
		WS-18	Wild boar 3/male/2–3 years	Adults	** + **	**−**
		WS-19	Wild boar 3/male/2–3 years	Adults	** + **	**−**
		WS-20 to WS-23	Wild boar 3/male/2–3 years	Adults	**−**	**/**
		WS-24	Wild boar 3/male/2–3 years	Nymphs	**−**	**−**
		WS-25	Wild boar 3/male/2–3 years	Adults	** + **	**−**
		WS-26	Wild boar 3/male/2–3 years	Adults	** + **	**−**
	Total	26	3		9	3
*H. longicornis*		WS-27	Wild boar 1/male/2–3 years	Adults	** + **	**−**
		WS-28	Wild boar 1/male/2–3 years	Adults	** + **	**−**
		WS-29	Wild boar 1/male/2–3 years	Nymphs	**−**	**/**
		WS-30	Wild boar 1/male/2–3 years	Nymphs	**−**	**/**
		WS-31 to WS-35	Wild boar 1/male/2–3 years	Adults	**−**	**/**
	Total	9	1		2	0

*JMTV* Jingmen tick virus,* qRT-PCR* quantitative reverse transcription-PCR

^a^JMTV RNA was detected in 9 *A. testudinarium* ticks and in 2 *H. longicornis* ticks; the partial S1 segment encoding nonstructural protein 1 (NSP1) of JMTV was obtained from only 3 *A. testudinarium* ticks (no *H. longicornis*) .

^b^Slash (∕) indicates that no experimental testing was carried out

Of the 35 tick specimens collected, positive qRT-PCR results for JMTV were obtained for both *A. testudinarium* and *H. longicornis *specimens. JMTV RNA was detected in 11 ticks (31.4%), including nine *A. testudinarium* ticks (9/26 [34.6%]) and two *H. longicornis* ticks (2/9 [22.2%]). However, the virus was not isolated from any positive tick samples.

### Phylogenetic analysis

**Fig. 2:**
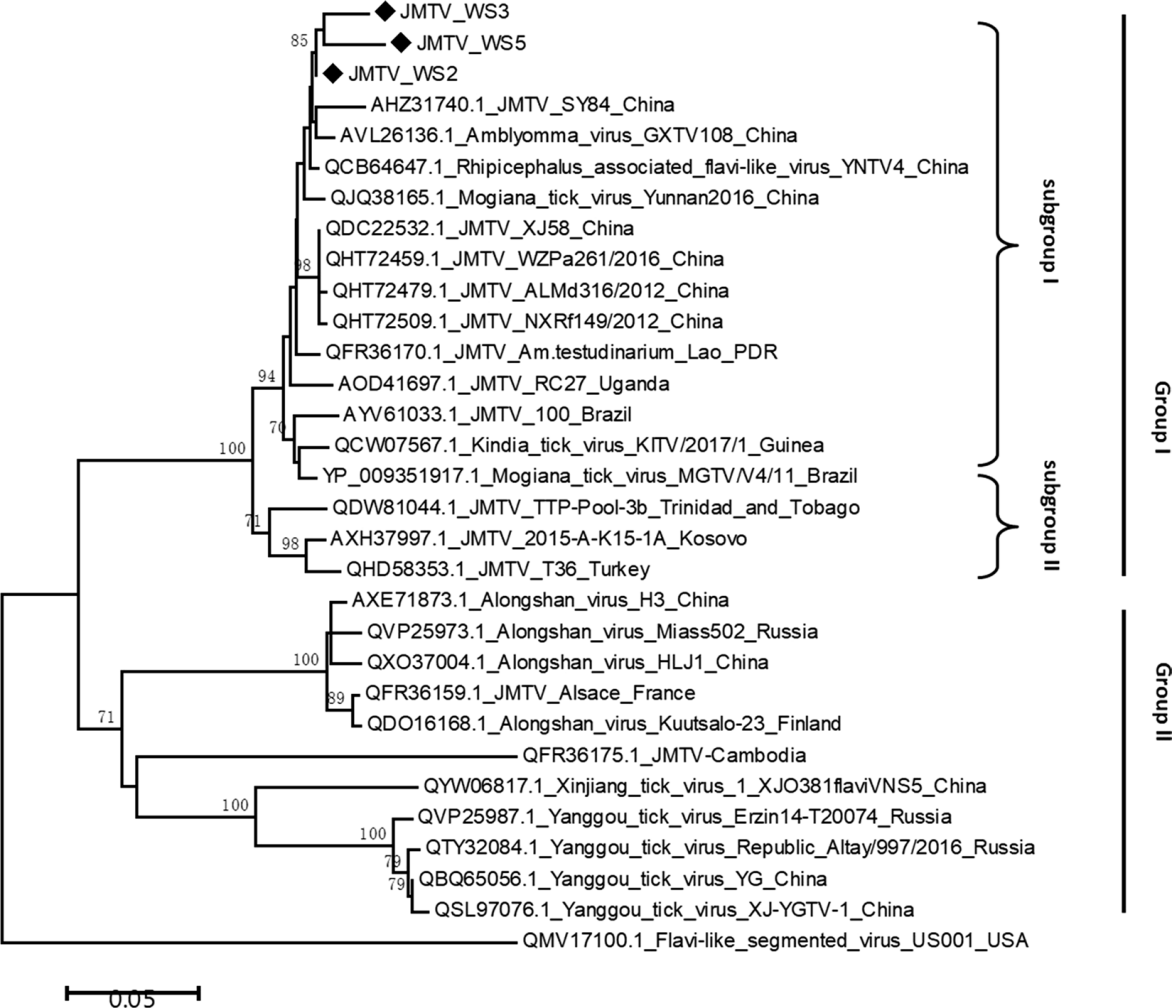
Phylogenetic analysis of the partial nucleotide sequences of JMTV_WS2, JMTV_WS3 and JMTV_WS5 with representative viruses of the Jingmenvirus group. The phylogenetic tree was built based on a 1071-bp fragment of the NSP1 gene and generated using a neighbor-joining method. The numbers above the branches indicate bootstrap values. Black rhombuses indicate JMTV sequences obtained in the current study. JMTV, Jingmen tick virus; NSP, nonstructural protein

In this study, the partial S1 segment encoding NSP1 of JMTV was successfully amplified and sequenced from three of the 11 ticks that tested positive for JMTV by qRT-PCR. The three positive ticks were all *A. testudinariu*. Pairwise distances analysis indicated that these three sequences shared a 98.4–99.3% nucleotide identity and had > 96% amino acid identity with each other. These results demonstrated that these sequences were extraordinarily closely related and possibly even descended from a single common ancestor. A phylogenetic tree was constructed based on three amino acid sequences of NSP1 obtained in this study, along with corresponding sequences of the Jingmenvirus group retrieved from the GenBank. As shown in Fig. 2, all JMTV-related sequences could be roughly divided into two major groups, with group I mainly containing JMTV from Asia, eastern Europe, Africa and Americas, Mogiana tick virus from Brazil and Kindia tick virus from Guinea, and group II comprising JMTV from France and Cambodia, ALSV and Yanggou tick virus from China, Finland and Russia. Within group I, viruses could be also basically classified into two subgroups. All JMTV-like strains from China, including JMTV_WS3 identified in this study, clustered with JMTVs from The Lao People’s Democratic Republic (Lao PDR) and Brazil in phylogenetic subgroup I, and JMTVs isolated from Kosovo, Turkey and Trinidad and Tobago formed subgroup II. The composition of subgroup I clearly revealed that the JMTVs identified to date in China was highly homologous. In particular, the three sequences in this study clustered together and exhibited a very close evolutionary relationship with JMTV_SY84 identified in *R. microplus* from Hubei Province. The results showed that these JMTV sequences obtained from Fujian Province were genetically close to the reported sequences from Hubei Province.

### Genome-wide analysis of JMTV

Direct next-generation sequencing (NGS) of the JMTV-positive tick sample (WS3) from Fujian Province provided 8.05 G bases data with total read numbers of 30.24 million. The complete nucleotide sequence of the four segments of the JMTV genome in this sample (WS-3) were obtained as: S1, 3115 bp; S2, 2845 bp; S3, 2824 bp; S4, 2795 bp. These sequences have been deposited in the GenBank database with accession numbers OM459837-OM459840.

**Fig. 3:**
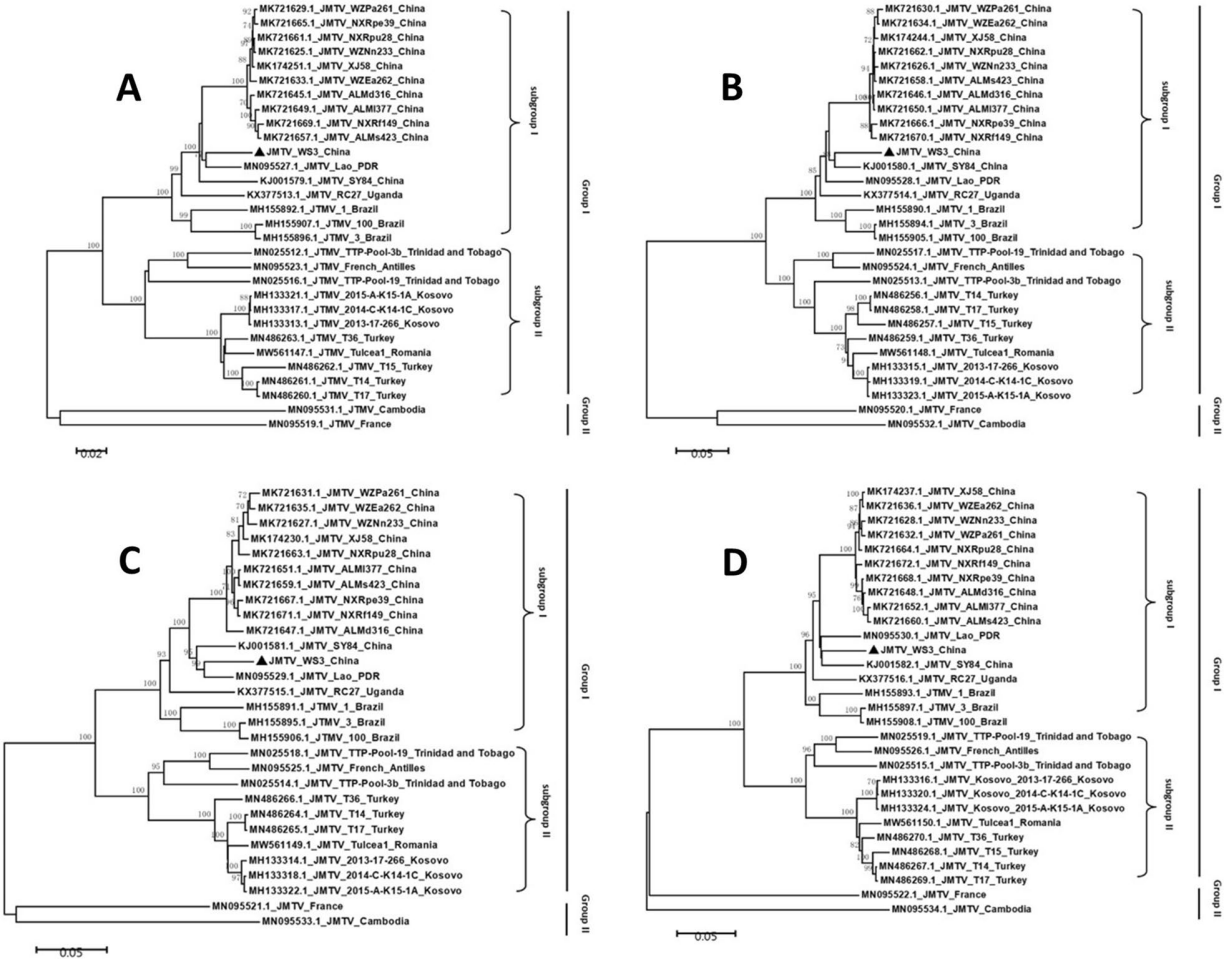
Phylogenetic analysis of the complete sequences of JMTV strains. JMTV_WS2 identified from ticks in this study (marked black with a triangle: **a** segment 1, **b** segment 2, **c** segment 3, **d** segment 4. Phylogenetic trees were constructed by the neighbor-joining method using MEGA 5.1 software. Bootstrap values are shown at the branches. GenBank accession numbers are listed for each strain/isolate/amplicon. Descriptions of the sequences published in GenBank were reproduced without changes

Compared with the information reported on the JMTV genome on the global scale, the JMTV sequences obtained in this study were shown to be similar to those found in Lao PDR, Uganda, Turkey, Kosovo, Brazil, Trinidad and Tobago and other regions of China, with a 76.9%-94.6% nucleotide identity, while relatively low nucleotide identity was found with the strains from France (57.1%-70.4%) and Cambodia (54.1%-70.5%). For a better analysis of these newly identified JMTVs from Fujian Province, phylogenetic trees were constructed using an neighbor-joining approach based on four complete segment sequences (Fig. 3). Despite exhibiting a high genetic diversity, all known JMTVs could still be classified into two major phylogenetic groups, with the first group comprising JMTVs identified from Asia, Africa, South America and Eastern Europe, and the second group containing viruses sampled from France and Cambodia. Within the first group, all JMTVs could be further divided into two subgroups. All Chinese strains, including the strains identified in this study, clustered together and formed subgroup I, together with the viruses identified in Lao PDR, Uganda and Brazil, while the strains from Trinidad and Tobago, Turkey, Kosovo and Romania constituted subgroup II. The JMTV_WS3 strain in this study had a close evolutionary relationship with the first subgroup and a further evolutionary relationship with the second subgroup and the second group containing Cambodia and France strains.

**Fig. 4:**
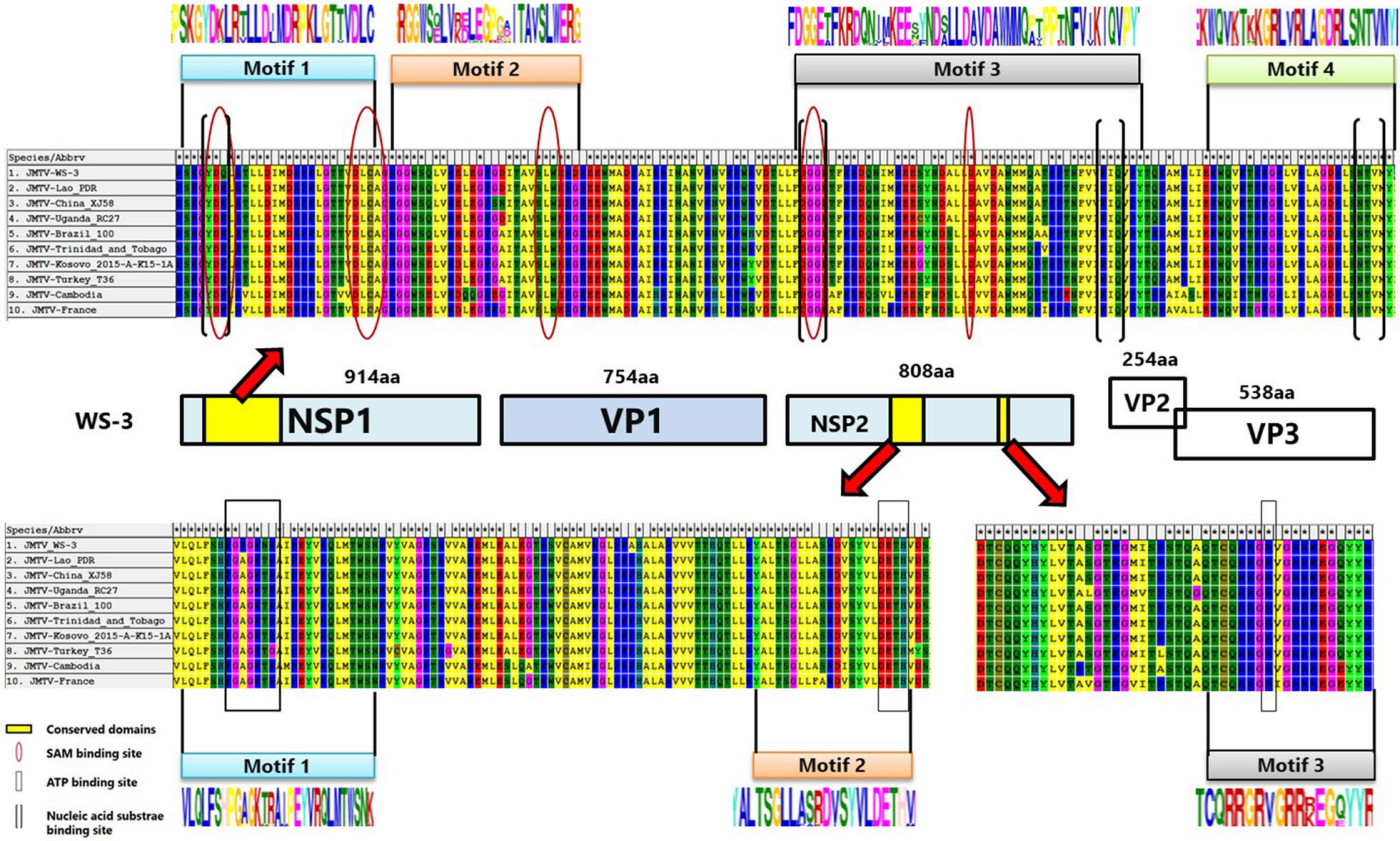
Comparative analysis of potential important protein binding sites of conserved domains confirmed on viral segments 1 and 3 of JMTV strains from different countries. Regions and residues corresponding to the conserved motifs of the virus are highlighted by different geometric shapes. aa, Amino acids; SAM, S-adenosylmethionine; VP, viral protein

The organization of the JMTV genome was much conserved among all available strains, including JMTV_WS3 mentioned in our study. Segment 1 encoding NSP1, which resembled *Flavivirus* NS5, manifested conserved domains for the S-adenosylmethionine (SAM) binding site and nucleic acid substrate binding site of a typical JMTV. Segment 3 encoded a unique ORF corresponding to another nonstructural protein (NSP2) with conserved domains for the ATP binding site, which presented homology with the NS2b-NS3 complex of flaviviruses. In addition, three structural proteins of JMTVs were encoded each in their own way, showing more diversity and unique properties in terms of their genetic organization. For example, glycoprotein was encoded by segment 2 in a monocistronic expression pattern, while segment 4 encoded capsid and membrane proteins in a bicistronic pattern and comprised two ORFs with overlapping reading frames in some strains. The amino acid sequences of JMTV strains from nine different countries were compared with the JMTV_WS3 reported in this study. Our results showed that little difference existed among the motifs in the conserved regions of JMTV nonstructural proteins (NSP1 and NSP2) from all over the world, and that the sequences of important protein binding sites were conservative, with almost no change (Fig. 4). These results demonstrated that *Flavivirus*-like protein of JMTV was very stable in nature and relatively slow to evolve, regardless of geographical location and environment.

## Discussion

Jingmen tick virus is a newly discovered segmented tick-borne RNA virus. Since the discovery of JMTV in 2014, our inherent understanding of the genomic structure and organization of flaviviruses has gradually changed. To our knowledge, JMTV is the first segmented RNA virus with a partial genome derived from unsegmented viral ancestors to be identified and reveals the possible evolutionary link between two viruses with different patterns of genome organization. Since its discovery, relevant reports on JMTV have been published across the globe. In addition, many viruses with similar genomes to JMTV, referred to as the Jingmenvirus group, have successively been found and confirmed, such as Mogiana tick virus (identified from ticks in Brazil [20]), Charvil virus (found in *Drosophila melanogaster* in the UK [21]), Wuhan flea virus (WHFV), Wuhan cricket virus (WHCV), Wuhan aphid virus 1 (WHAV1), Wuhan aphid virus 2 (WHAV2) and Yanggou tick virus (from ticks in China [22]), Guaico Culex virus (from mosquitoes in Trinidad, Peru, and Panama [23]) and ALSV (from ticks and humans in China, Finland and Russia [3, 24, 25]). These findings show that there is a large class of viruses with similar genomic structure in nature. Jingmenvirus is considered to be a large group of segmented RNA viruses that are phylogenetically linked with unsegmented viral ancestors. With the increasing number of members of the Jingmenvirus group being identified, this group of viruses has been designated an independent branch and became the focus of current research [15]. Fujian Province is rich in forest resources and diverse in insect flora and species distribution, factors which support the circulation of numerous arthropod-borne viruses. The tick specimens used in the present study were collected from wild boars far away from human activities, and the results could reflect the survival of this virus in the wild environment to a great extent.

In this study, JMTV was identified for the first time in Fujian Province, demonstrating that JMTV may be widely distributed throughout southeastern China and form a stable ecological cycle in the local area. By comparison with a large-scale survey of JMTV in ticks from Turkey [14], the JMTV-positive rate in ticks in the present study was much higher (31.4%). This higher positivity rate may be due to the differences in sample sizes and sampling methods, or it could be that the ticks had ingested virus from the host. However, our results are consistent with those of earlier surveys conducted in Zhejiang Province (China) and in the French Antilles [13, 16], possibly due the sampling method of collecting engorged ticks from animals in all of these studies. It would appear probable that the risk of JMTV transmission among ticks could be increased by co-feeding in animals.

The sequence of the complete JMTV genome were obtained by direct NGS from one tick (WS-3) which tested positive for JMTV by qRT-PCR. This sequence was compared with all available JMTV sequences in the GenBank database. The genome topology and functional organization of JMTV_WS3 from Fujian Province were identical to those from other regions, with the highest sequence identities to JMTV strains from Lao PDR and China and the lowest to strains from France and Cambodia. This result is in agreement with the results of phylogenetic analysis on all four genomic fragments. Thus, a spatial segregation of viral subclades was observed, signifying the current local adaptive processes of JMTV. A comprehensive analysis of the amino acid sequences and conserved regions of all available JMTV was also performed. JMTV_WS3 shared significant similarities with other strains from different regions of China. In addition, insignificant heterogeneity were also observed in important protein binding sites of conserved regions, such as the SAM binding site and nucleic acid substrate binding site in segment 1 and ATP binding site in segment 3, among these viruses from different continents. These results demonstrate that JMTV is widely distributed over the world and that is has adapted well to the local natural ecological resources, showing a certain diversity in terms of its genome. However, an obvious homogeneity and low speed of evolution were apparent among the core functional sites of some important proteins.

Unlike the common tick species collected from the body surface of animals in previous research, *A. testudinarium* was the dominant tick species in this study. This tick mainly lives in mountain forests or fields and is widely distributed throughout the coastal areas of southeastern China. Its main host animals are large wild mammals and livestock, which also invade humans. The peak season of its annual activities is from April to October [19, 26]. Interestingly, JMTV was first identified in *A. testudinarium*, which extended the tick-borne infection spectrum of JMTV and increased the diversity of virus hosts or vectors. Our results shed a new light on our understanding of the survival mode of this virus in nature and its life-cycle characteristics. So far, JMTV has been documented to cause human diseases and viremia in some individuals [27, 28], but systemic and detailed research is lacking. Hence, the potential pathogenic of JMTV to human or animals deserves consistent attention. Unfortunately, we were unable to successfully isolate viable virus in the present study, possibly due to the low copy number of live virus in these ticks or inappropriate cell lines.

A phylogenetic tree was built by neighbor-joining method based on the partial amino acid sequences of segment S1 amplified in this study and other sequences of Jingmenvirus group from GenBank. The phylogenetic analysis demonstrated that the three strains of JMTV obtained in this study had the closest relationship with isolate SY84 found in Hubei Province [12] and that they were very similar to each other. In addition, all Chinese strains, including those identified in this study, clustered with those from Lao PDR and formed a separate clade, illustrating that these strains in specific geographical locations manifested a consistent evolutionary direction under similar environmental factors, or that they shared a common virus ancestor. In our opinion, JMTV is widely distributed in eastern and southern Asia and highly conserved in evolutionary terms as an natural focus virus. However, the sequences of JMTV_WS2, JMTV_WS3 and JMTV_WS5 were distinct from previously characterized viruses in France and Cambodia, possible due to the evolutionary consequences of different JMTV lineages or a wide range of mutations in the genome. In addition, the sequences of JMTV in this study were remotely different from other members of the Jingmenvirus group, such as ALSV and Yanggou tick virus identified in arthropods both inside and outside of China. These data suggest that the group had a large number of members and that the gene recombination between flavivirus and other segmented RNA viruses was very common. From another perspective, this also reflects that gene swapping occurs not only between segmented viruses, but also between unsegmented and segmented viruses. Up to now, the precise recombination mechanism and detailed evolutionary characteristics of the JMTV genome remain to be further illustrated, and it is possible that this segmented flavivirus has a complex evolutionary history.

## Conclusions

In conclusion, our study provides important molecular evidence on JMTV in a non-endemic region of southeastern China. We report here the first discovery of this virus in *A. testudinarium* attached to a wild animal, possibly signifying that JMTV is likely to be able to establish a stable life-cycle in the local wild environment through wild animals and tick vectors. As such, it poses a specific health threat to local residents. In short, research on JMTV is still in the initial stage, and further studies are required to elucidate the pathogenicity of this virus to animals and its epidemiological characteristics in nature.

## Supplementary Information

**Additional file 1**. Conventional PCR primers for complete genome sequences of JMTV_WS3

## Data Availability

Data will be available on request by email to the corresponding author. All the nucleotide sequences were deposited in GenBank under accession numbers OM363694-OM363695, OM459837-OM459840.

## Abbreviations

*COI*: Cytochrome* c* oxidase I
JMTV: Jingmen tick virus
NGS: Next-generation sequencing
NSP: Nonstructural protein
qRT-PCR: Quantitative reverse transcription-PCR
ORF: Open reading frame
RACE: Rapid amplification of cDNA end
SAM: S-Adenosylmethionine
