# Supplementary material for: Identification of Jingmen tick virus (JMTV) in Amblyomma testudinarium from Fujian Province, southeastern China
Source: Parasit Vectors. 2022 Sep 27;15:339. doi: 10.1186/s13071-022-05478-2 (PMC9513871; doi:10.1186/s13071-022-05478-2)
Supplement: Supplementary file 1 — Additional file 1. Conventional PCR primers for complete genome sequences of JMTV_WS3 [file 13071_2022_5478_MOESM1_ESM.docx]

Conventional PCR primers for complete genome sequences of JMTV_WS3:

JMTV S1-F1:gttaaaagaggccgccctttact

JMTV S1-R1:atcatccatgcgtccacagc

JMTV S1-F2:gccctggcgtgtagacacact

JMTV S1-R2:atattgaacacggctcgaggtg

JMTV S1-F3:ggatcagaaggcacgcga

JMTV S1-R3:ccatttcttcatcctccgctagt

JMTV S1-F4:aagaagtcaatggacgctaccg

JMTV S1-R4:gagccagcattctgcattctg

JMTV S1-F5:cagtcagggacgtgggaga

JMTV S1-R5:cctctttagtaacttgcagatcagc

JMTV S2-F1:gtttaaaaagcggaccgtgc

JMTV S2-R1:cccgtgggctcagagtagtc

JMTV S2-F2:cctctgggaccaggctctc

JMTV S2-R2:ggtcgggccacacctggt

JMTV S2-F3:cagcatgcctcactggctct

JMTV S2-R3:acaaggccgagcccaatg

JMTV S2-F4:gggtggcttggttgaagaact

JMTV S2-R4:cctgcagtgtggatgctacgt

JMTV S2-F5:ggagagtcacccatggtttgat

JMTV S2-R5:gtgttcccttccggatcctc

JMTV S3-F1:gttaaaaagcgccagctgagg

JMTV S3-R1:ctacactaggctcatacctgcggt

JMTV S3-F2:catatcggaccatgggcct

JMTV S3-R2:gtttgatgtgttgtgacgactactct

JMTV S3-F3:acctggtccaacaaggtctacgt

JMTV S3-R3:gaagccgtgacaaggtagtggt

JMTV S3-F4:acagtggtcttgagtaggaagacgtat

JMTV S3-R4:atcttccattctctcccatctaacg

JMTV S3-F5:gatcaggtgtacaggtggctgac

JMTV S3-R5:gtagactagcgaccggctgg

JMTV S4-F1:gtttaaaaacggccgccct

JMTV S4-R1:tcagtgtaggtctttccgccag

JMTV S4-F2:cctgatcacaggagaggatgttct

JMTV S4-R2:gccgaatccagcggtacat

JMTV S4-F3:atgaccatcgcgggagatt

JMTV S4-R3:ccaggttgtagacctgatccatct

JMTV S4-F4:ctacatcgcgtcttcctttgatgt

JMTV S4-R4:tgccaatcctgctgtgtattgt

JMTV S4-F5:ttatgtcatacctgagagggactaacg

JMTV S4-R5:gttgtcaactccggagtatggagt
